# Supplementary material for: Hepatitis B Core-Related Antigen Is Useful for Predicting Phase and Prognosis of Hepatitis B e Antigen-Positive Patients
Source: J Clin Med. 2022 Mar 21;11(6):1729. doi: 10.3390/jcm11061729 (PMC8956075; doi:10.3390/jcm11061729)

Supplementary Table S1. Baseline characteristics of HBeAg-positive and negative patients

Variables	HBeAg positive patients (n=243, 62.8%)	HBeAg negative patients (n=144, 37.2%)	*P* value
Age, years	37.6 ± 12.4	46.0 ± 10.3	<0.001
Male, n (%)	160 (65.8)	100 (69.4)	0.466
Diabetes, n (%)	10 (4.1)	13 (9.0)	0.048
Platelet count, $\times 10^9$/L	187.9 ± 57.1	167.2 ± 48.4	<0.001
PT INR	1.06 ± 0.09	1.07 ± 0.10	0.090
Alanine aminotransferase, IU/L	195.0 ± 230.8	150.8 ± 245.3	0.076
Bilirubin, mg/dL	0.77 ± 0.48	0.93 ± 1.98	0.218
Albumin, g/dL	4.0 ± 0.7	4.0 ± 0.4	0.802
AFP, ng/mL	15.7 ± 54.3	10.5 ± 29.9	0.302
HBV DNA, log IU/mL	7.3 ± 1.2	5.2 ± 2.0	<0.001
HBsAg, log IU/mL	6.9 ± 0.6	5.1 ± 2.6	<0.001
HBcrAg, log U/mL	7.7 ± 1.0	5.1 ± 1.5	<0.001

Variables are expressed as mean ± standard deviation or n (%). HBeAg, hepatitis B e antigen; INR, international normalized ratio; HBV, hepatitis B virus; HBsAg, hepatitis B surface antigen; HBcrAg, hepatitis B core-related antigen

Supplementary Table S2. Predictors for inactive phase in HBeAg-negative patients

Variables	Rating	Univariate	Multivariate		
		P value	Hazard ratio	95% confidence interval	P value
Age	years	0.174			
Male sex		0.667			
Diabetes	0=no; 1=yes	0.807			
Platelet count	$\times 10^9$/L	0.010	1.009	0.997-1.022	0.152
PT INR		0.370			
Alanine aminotransferase	IU/L	0.196			
Bilirubin	mg/dL	0.301			
HBV DNA	log IU/mL	<0.001	0.643	0.456-0.905	0.011
HBsAg	log IU/mL	0.858			
HBcrAg	log U/mL	0.001	0.735	0.438-1.234	0.244

HBeAg, hepatitis B e antigen; INR, international normalized ratio; HBV, hepatitis B virus; HBsAg, hepatitis B surface antigen; HBcrAg, hepatitis B core-related antigen

Supplementary Figure S1. Flowchart of patients

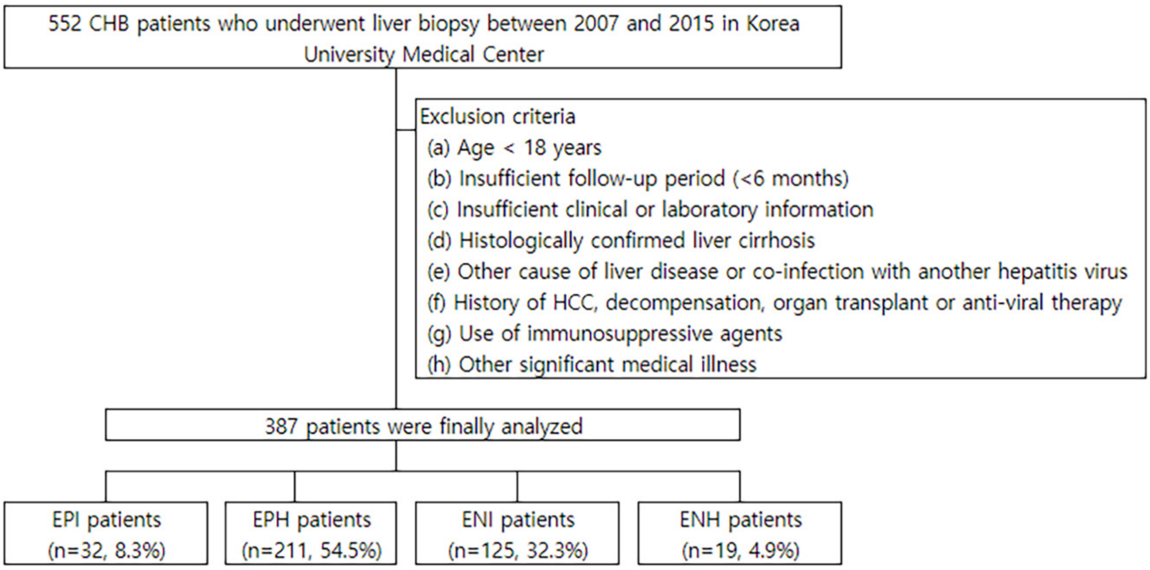

Supplement: Supplementary file 1 [file jcm-11-01729-s001.zip › jcm-1627584-supplementary.pdf]
